# It started with a Cys: Spontaneous cysteine modification during cryo-EM grid preparation

**DOI:** 10.3389/fmolb.2022.945772

**Published:** 2022-08-05

**Authors:** David P. Klebl, Yiheng Wang, Frank Sobott, Rebecca F. Thompson, Stephen P. Muench

**Affiliations:** ^1^ School of Biomedical Sciences, Faculty of Biological Sciences & Astbury Centre for Structural and Molecular Biology, University of Leeds, Leeds, United Kingdom; ^2^ School of Molecular and Cellular Biology, Faculty of Biological Sciences & Astbury Centre for Structural and Molecular Biology, University of Leeds, Leeds, United Kingdom

**Keywords:** single particle cryo-EM, sample preparation, air–water-interface, protein modification, structure determination

## Abstract

Advances in single particle cryo-EM data collection and processing have seen a significant rise in its use. However, the influences of the environment generated through grid preparation, by for example interactions of proteins with the air-water interface are poorly understood and can be a major hurdle in structure determination by cryo-EM. Initial interactions of proteins with the air-water interface occur quickly and proteins can adopt preferred orientation or partially unfold within hundreds of milliseconds. It has also been shown previously that thin-film layers create hydroxyl radicals. To investigate the potential this might have in cryo-EM sample preparation, we studied two proteins, HSPD1, and beta-galactosidase, and show that cysteine residues are modified in a time-dependent manner. In the case of both HSPD1 and beta-galactosidase, this putative oxidation is linked to partial protein unfolding, as well as more subtle structural changes. We show these modifications can be alleviated through increasing the speed of grid preparation, the addition of DTT, or by sequestering away from the AWI using continuous support films. We speculate that the modification is oxidation by reactive oxygen species which are formed and act at the air-water interface. Finally, we show grid preparation on a millisecond timescale outruns cysteine modification, showing that the reaction timescale is in the range of 100s to 1,000s milliseconds and offering an alternative approach to prevent spontaneous cysteine modification and its consequences during cryo-EM grid preparation.

## Introduction

In the past decade, cryo-EM has become one of the major techniques for protein structure determination with rapid improvements in microscope hardware and data processing software, recently reaching atomic resolution (Nakane et al., 2020; Yip et al., 2020). However, the physical and chemical processes impacting macromolecules during cryo-EM grid preparation are not yet fully understood and sample preparation for cryo-EM has increasingly become a bottleneck (Weissenberger et al., 2021). Conventional cryo-EM sample preparation is done by manually applying a small volume of sample (typically ∼3 µl) to a grid. Excess liquid is then blotted away with filter paper in an automated fashion, leaving a thin liquid film suspended over the grid foil holes only 10s to 100s nm thick. After blotting, the grid with the thin film is plunged into liquid ethane, where the sample is vitrified. The blotting process usually takes seconds, as does the manual sample application process. While undeniably successful for many samples, this approach is not universally successful, in part due to problems arising from air–water interface (AWI) interactions.

The vast majority of proteins are thought to interact with the AWI, which for some samples may affect particle orientation (Noble et al., 2018a) and in some cases the integrity of the sample (D'Imprima et al., 2019). In sample films with suitable thickness for cryo-EM, local protein unfolding at the AWI can occur in 100s of milliseconds (Klebl et al., 2020) and can potentially be detrimental to accurate structure determination or interpretation. Effects of the AWI on orientation or resolution can be reduced by minimizing the time the sample spends in the thin film environment (Noble et al., 2018b; Levitz et al., 2021), and by other approaches for example the use of surfactants such as detergents (Chen et al., 2019). Solutions to preferred orientation or denaturation are often protein-specific, and there is no one solution that fits all (Drulyte et al., 2017). Charge and hydrophobicity have been identified as important factors that govern protein-AWI interactions (Glaeser, 2018; Li et al., 2021) but there may be other effects.

Properties of the AWI other than charge or hydrophobicity have received less attention in the cryo-EM field, but are likely to also influence the protein sample and have been studied for example in the fields of catalysis or atmospheric chemistry. Most research has been done using microdroplets but many factors also apply to the AWI of a thin film. The pH at or near the AWI is thought to be different from bulk solution (Buch et al., 2007; Wei et al., 2018) and it has also been suggested that a very large electric field exists that is present at the AWI, on the order of 10^8^–10^10^ V/m^−1^ (Kathmann et al., 2008). This electric field may be sufficient to split water into OH radicals which would be able to modify the protein directly. Moreover, this environment has been shown to spontaneously produce H_2_O_2_ from pure water when atomizing into small droplets at an approximate concentration of 30 μM (Lee et al., 2019). The downstream effects that may result from the production of H_2_O_2_ in combination with other negative features of the AWI are poorly understood but those residues more sensitive to oxidation could become modified. Moreover, even more, complex reactions such as peptide bond formation have been shown to occur at the AWI (Griffith & Vaida, 2012) and some enzymes exhibit a dramatic increase in reaction rates in the presence of a large AWI (Zhong et al., 2020).

The present work examines cysteine residues and shows evidence for their modification during cryo-EM grid preparation, which is prevented either by adding reducing agents such as DTT, by sequestering away from the AWI, or by fast grid preparation. This suggests that the modification is dependent on exposure within the thin film and/or time at the AWI. Conformational changes are associated with cysteine modification and observed in both the model proteins used for this study, HSPD1, and β-gal, albeit to different degrees. We hypothesize that free cysteines are modified by hydroxyl radicals from the AWI before exposure of the sample to the electron beam and that this process may occur in many cases but has been overlooked because unambiguous identification requires high-resolution reconstructions and multiple datasets.

## Materials and methods

HSPD1 was expressed and purified as previously described (Klebl et al., 2021). Briefly, 4 h after induction [250 µM IPTG (final concentration)], bacterial cells were harvested by centrifugation (10 min, 4,000 rpm). Cell pellets were resuspended in 20 ml lysis buffer (50 mM Tris, 500 mM NaCl, 10 mM imidazole pH 8), containing 1 mM PMSF and protease inhibitor cocktail (set V, Calbiochem). Sonication was performed after further resuspending the cells in a Dounce homogenizer, Centrifugation was used to clear lysate (30 min, 17,000 rpm) with the resultant supernatant applied to 3.5 ml Ni-NTA resin (equilibrated in lysis buffer). The resin was washed with 2 × 100 ml lysis buffer and 100 ml of 50 mM Tris, 300 mM NaCl, and 50 mM imidazole (pH 8). Elution was achieved using 10 ml of 50 mM Tris, 300 mM NaCl, and 300 mM imidazole (pH 8). Protein was cleaved with TEV protease (1.6 mg per 1 L of culture and 1 mM DTT) for 4 h at RT. The cleaved protein was subsequently dialyzed overnight into 50 mM Tris, 150 mM NaCl pH 7.5 (2 L) using 10 kDa molecular weight cutoff (MWCO) dialysis tubing with the resultant product incubated with 1.5 ml Ni-NTA resin (pre-equilibrated in lysis buffer) for 1 h. Flow-through was then concentrated to 20–30 mg/ml in a Sartorius, 10 kDa MWCO spin concentrator. For reconstitution into oligomers, monomeric HSPD1 was mixed with 100 µl KCl (1 M), 100 μl Mg(OAc)_2_ (1 M), and 400 μl Mg-ATP (50 mM, pH 7) and incubated for 90 min at 30°C. The sample was then centrifuged at 13,000 rpm for 10 min at RT, with the resultant supernatant loaded onto a HiLoad 16/600 Superdex 200 gel filtration column (GE Healthcare) in 50 mM Tris, 300 mM NaCl, 10 mM MgCl_2_ (pH 7.7).

Concentrated HSPD1 was diluted to the target concentration in the desired buffer. β-gal was obtained from Sigma-Aldrich (G6008). As a final purification step before grid preparation, β-gal was purified by size-exclusion chromatography using a Superose 6 10/300 Increase column in 25 mM Tris, 50 mM NaCl, 2 mM MgCl_2_ pH 8. The peak eluting at 14 ml was collected, concentrated to 4.4 mg/ml using a 30 kDa cut-off spin concentrator, and diluted to the target concentration in the desired buffer.

Conventional blotted grids of HSPD1 (“blotted,” “5 mM Met,” and “1 mM Asc”) were prepared using a Vitrobot at > 90% humidity and 20–22°C temperature (blot force 6, blot time 6 s). Quantifoil 300 mesh Cu R1.2/1.3 grids were used after glow discharge in a Cressington 208 carbon coater with a glow discharge unit (10 mA, 30 s, 0.1 mbar). The final protein concentration was 2.3 mg/ml, the buffer was 25 mM Tris, 150 mM NaCl, 5 mM MgCl_2_ pH 7.7. The “5 mM Met” sample additionally contained 5 mM L-methionine, and the “1 mM Asc” sample additionally contained 1 mM ascorbic acid.

Conventional blotted grids of β-gal (“blotted,” “1 mM DTT,” and “1 mM TCEP”) were prepared using a Vitrobot at > 95% humidity and 4–6°C temperature (blot force 2, blot time 6 s). Quantifoil 300 mesh Cu R1.2/1.3 grids were used after glow discharge in a Cressington 208 carbon coater with a glow discharge unit (15 mA, 30 s, 0.1 mbar). The final protein concentration was 0.25 mg/ml for “blotted” β-gal and 1 mg/ml for “1 mM DTT”/“1 mM TCEP” β-gal. The buffer was in 25 mM Tris, 50 mM NaCl, and 2 mM MgCl_2_ pH 8. The “1 mM DTT” sample additionally contained 1 mM DTT, and the “1 mM TCEP” sample additionally contained 1 mM TCEP.

“Fast” grids were prepared using an in-house device for fast grid preparation as previously described (Klebl et al., 2020). Briefly, this device works by depositing small- and high-velocity droplets of the sample onto a fast-moving grid which is subsequently plunged in liquid ethane to produce vitreous ice. By removing the traditional “blotting” stage the process from spray to vitrification can be achieved in >5 ms. Changes in the nozzle to grid distance, plunge speed, and liquid flow rate can be used to modify the speed of grid making. Self-wicking grids supplied by SPT Labtech were used, after glow discharge in a Cressington 208 carbon coater with a glow discharge unit (15 mA, 80 s, 0.1 mbar) to assist with droplet spreading on the grid. The spray nozzle/ethane distance was 2.2 cm, the nozzle/grid distance was 1.2 cm, and the spray gas pressure was 2 bar. For “fast” HSPD1, a liquid flow rate of 7.8 μl/s was used. The plunge speed was 1.5 m/s and a final protein concentration of 5 mg/ml was used in 25 mM Tris, 150 mM NaCl, and 5 mM MgCl_2_ pH 7.7. For “fast” b-gal, a liquid flowrate of 5.2 μL/s was used. The plunge speed was 1.3 m/s and a final protein concentration of 4.4 mg/ml was used in 25 mM Tris, 50 mM NaCl, and 2 mM MgCl_2_ pH 8.

The main parameters for data collection and processing are listed in Supplementary Tables S1, S2. All single-particle datasets were collected using a Titan Krios G2 microscope using Thermo Fisher Scientific EPU software (Thompson et al., 2019). The “5 mM Met” and “1 mM Asc” HSPD1 datasets were collected with a Falcon III detector in integrating mode. The “fast” β-gal dataset was collected with a Falcon IV detector in counting mode and a Selectris energy filter set to a width of 10 eV. All other datasets were collected with a Falcon IV detector in counting mode. Data processing pipelines for HSPD1 are shown in Supplementary Figures S2−S5. Data processing pipelines for β-gal are shown in Supplementary Figures S6−S10. Most processing steps were carried out using RELION 3.1, and in some cases, non-uniform refinement in cryoSPARC was used (Zivanov et al., 2018; Punjani, Zhang, & Fleet, 2020). The beam-induced motion was corrected using MotionCor2, in some cases with RELION’s implementation, and CTF-estimation was done using gctf or CTFFIND (Rohou & Grigorieff, 2015; Zhang, 2016; Zheng et al., 2017).

Docking of atomic models and visualization of maps and models was done in Chimera or ChimeraX (Pettersen et al., 2004; Goddard et al., 2018). Small adjustments to docked models, where needed were done using Isolde within ChimeraX (Croll, 2018).

## Results

**FIGURE 1:**
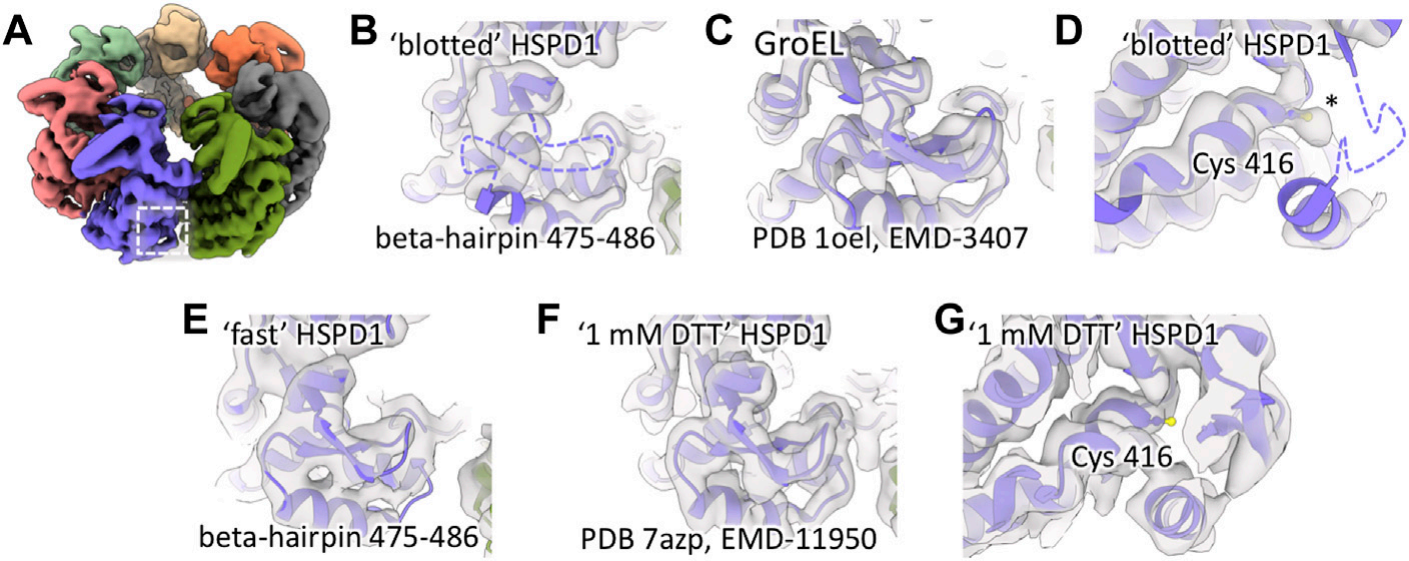
Local unfolding and additional density for Cys416 of HSPD1. **(A)** Reconstruction of “blotted” HSPD1 (determined to 3.4Å) with the region around beta-hairpin 475–486 highlighted by the box. **(B)** Beta-hairpin 475–486 is unfolded in “blotted” HSPD1, the unresolved region is indicated with a dashed purple line. **(C)** Equivalent region in GroEL (determined to 3.3Å), showing an intact beta-hairpin. **(D)** Additional density for Cys416 in “blotted” HSPD1, indicated by an asterisk. **(E)** “Fast” HSPD1 (determined to 6.8Å) has density indicating an intact beta-hairpin 475–486. **(F)** “1 mM DTT” HSPD1 (determined to 3.5Å) shows an intact beta-hairpin 475–486 and **(G)** no additional density for Cys416.

Our previous structural studies of the mitochondrial chaperonin HSPD1 (Hsp60) (Klebl et al., 2021) included cryo-EM data collection in a range of buffers and using different grid-preparation approaches. As a part of this, we prepared cryo-EM grids of HSPD1 in 25 mM Tris, 150 mM NaCl, and 5 mM MgCl_2_ pH 7.7 and obtained a reconstruction at 3.4Å resolution by standard single-particle data processing methods. In this cryo-EM map (“blotted” HSPD1), we noticed broken and weak density for a surface-exposed beta-hairpin near the ATP-binding site of the protein, suggesting that the region is disordered (residues 475–486, Figures 1A, B) while it is well resolved in cryo-EM structures of the bacterial homolog GroEL (Figure 1C) (Joseph et al., 2016). A more detailed inspection of this region of HSPD1 showed unexpected density for cysteine 416 near the disordered segment, suggesting that Cys416 may be chemically modified (Figure 1D). The map resolution was not sufficient to identify the modification but we noticed that the additional density of HSPD1 cysteine 416 would sterically clash with an intact beta-hairpin 475–486, so we concluded that Cys416 modification is linked to beta-hairpin 475–486 unfolding. The equivalent amino acid residue in GroEL is an alanine, which cannot undergo the same modifications and could explain why this region of GroEL shows different behavior.

Using data from a previous study (Klebl et al., 2020), we compared our “blotted” HSPD1 to reconstructions from fast grid preparation with delays of less than 100 ms between sample application and vitrification. These reconstructions of HSPD1 (EMD-10883, EMD-10884, EMD-10885) in contrast to the blotted grids, did not show signs of beta-hairpin 475–486 unfolding. To confirm this, we prepared new grids of HSPD1 in the same buffer as ‘blotted’ HSPD1 using our in-house setup for fast grid preparation with a delay of 14 ms between sample application and vitrification. Cryo-EM data collection and processing gave a reconstruction at 6.8 Å resolution of “fast” HSPD1. This reconstruction, like previous cryo-EM maps from a fast grid preparation approach, showed density for beta-hairpin 475–486 and no sign of local unfolding (Figure 1E). Despite the limited resolution, we concluded that Cys416 was unmodified in “fast” HSPD1 because we expect the Cys416 modification to be incompatible with an intact beta-hairpin 475–486.

We hypothesized that Cys416 modification in “blotted” HSPD1 was oxidation, and that this was the cause for local unfolding. Consequently, the addition of a reducing agent should alleviate oxidation. We added 1 mM DTT to the buffer before conventionally blotting grids (Klebl et al., 2021). In line with our hypothesis, this “1 mM DTT” HSDP1 showed a well-resolved beta-hairpin 475–486 and no signs of modification of Cys416 (Figures 1F, G).

Together, the data show that with fast grid preparation or DTT added, HSPD1 beta-hairpin 475–486 is structurally ordered. When using conventional blotting in the absence of additives, however, Cys416 appears to be modified and beta-hairpin 475–486 disordered. We concluded that oxidation and unfolding occur during grid preparation in the thin-film environment at the air-water interface (AWI). We further reasoned that if exposure to the AWI is the cause for Cys416 oxidation, this spontaneous modification during grid preparation should not be specific to HSPD1 and surface-exposed cysteines in other proteins may also be affected.

**FIGURE 2:**
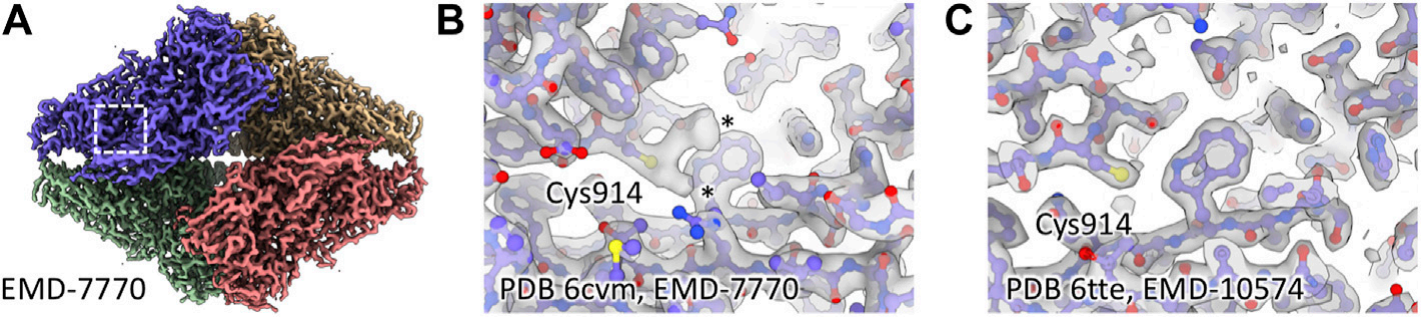
Additional cysteine density in published β-gal structures. **(A)** Region around Cys914 in EMD-7770. **(B)** Cys914 shows additional density in EMD-7770 (1 mM TCEP), marked with asterisks. **(C)** There is no additional density for Cys914 in EMD-10574 where 5 mM DTT was present during grid preparation.

To understand whether cysteine oxidation was a more general phenomenon during cryo-EM grid preparation, we searched the literature and electron microscopy database (EMDB) for unexpected cysteine modifications. Interestingly, we found cases that indicate modified cysteines in other cryo-EM density maps. In an atomic resolution reconstruction of human apoferritin, the authors identified a sulfenic acid group on Cys90 (Yip et al., 2020). We also found additional density for cysteine residues in published structures of beta-galactosidase (β-gal) (Bartesaghi et al., 2018; Saur et al., 2020), while there were no additional densities in a different structure when the buffer contained DTT (Figures 2A–C).

**FIGURE 3:**
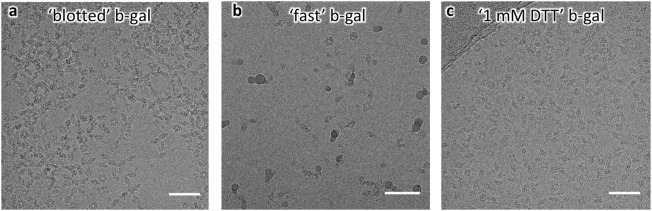
Raw micrographs of β-gal. **(A)** “Blotted” β-gal shows aggregated particles. **(B)** “Fast” β-gal shows lower apparent concentration and dispersed particles. **(C)** “1 mM DTT” β-gal shows well-dispersed particles at high concentration.

Encouraged by these findings, and given the relative amenability of β-gal to single-particle cryo-EM analysis we prepared grids by blotting, in the absence of additives (25 mM Tris, 50 mM NaCl, and 2 mM MgCl_2_ pH 8) or with 1 mM DTT added to the buffer. Furthermore, β-gal grids were prepared by fast grid preparation with a time delay of 18 ms between sample application and vitrification. Striking differences between the grid-preparation conditions were apparent directly from raw images: In the absence of DTT, particles were visibly aggregated and the concentration had to be lowered to give useable cryo-EM images (0.25 mg/ml, Figure 3A). Grids prepared at high speeds generally require higher protein concentration, but most particles were well dispersed in the raw images (Figure 3B), suggesting that reduced exposure to the AWI reduced aggregation. With 1 mM DTT in the buffer, particles were well dispersed at high concentrations (1 mg/ml used for grid preparation, Figure 3C).

**FIGURE 4:**
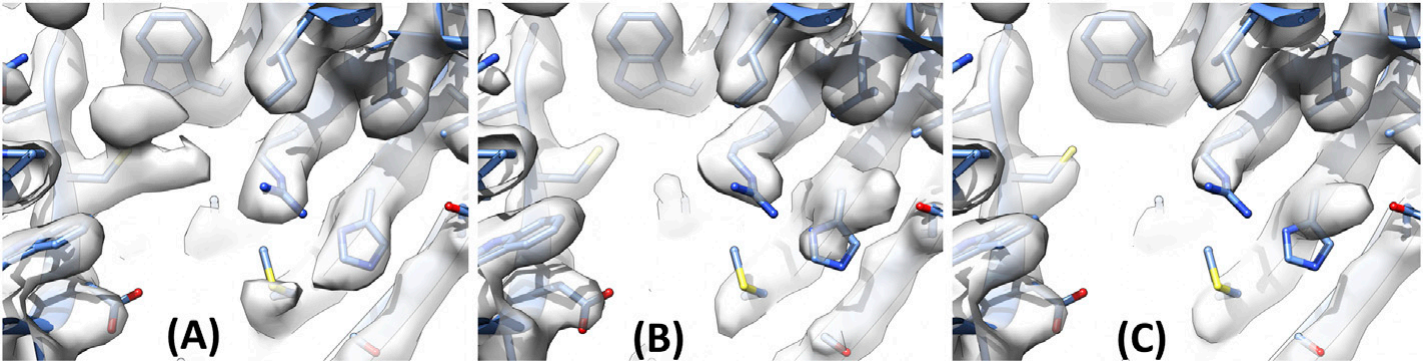
Β-gal Cysteine 914 and Histidine 653 under different grid preparation conditions. **(A)** “Blotted” β-gal shows additional density for Cys914. **(B)** “Fast” β-gal and **(C)** “1 mM DTT” β-gal show no additional density for Cys914. His653 adopts a different rotamer in “blotted” β-gal **(A)** where a modification is seen on Cys914, compared to **(B)**,**(C)**.

Data collection and processing for the three beta-galactosidase grids (“blotted”, “fast”, “1 mM DTT”) resulted in reconstructions at 2.2, 2.4, and 2.1 Å, respectively. As expected, the three reconstructions appeared very similar overall, with well-resolved side-chains. However, the density for Cys914 differed between “blotted” and “1 mM DTT”/“fast” β-gal (Figures 4A–C). There was clear additional density for Cys914 in “blotted” β-gal (Figure 4A) while in “fast” and “1 mM DTT” β-gal Cys914 showed no additional density. Interestingly, His653 which is located near Cys914 adopted a different rotamer in “blotted” β-gal than in “fast”/“1 mM DTT” β-gal (Figure 4). This may be caused by modification of Cys914, and by subtle changes propagated through the structure or solvent to His653. The bidentate shape of the density for Cys914 in “blotted” β-gal may be consistent with a sulfinic acid group, but the unambiguous assignment of the modification would require higher resolution. We also investigated if any other residues were modified in β-gal and found two further Cys residues (77 and 248), which also display a similar trend to that seen for 914 (Supplementary Figure S1). Other Cys residues remained unmodified, indicating that the local environment and/or surface exposure may also play a role in the propensity for modification at the Cys residue.

**FIGURE 5:**
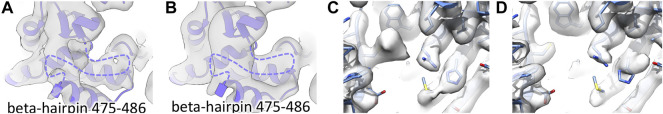
Effects of other radical scavengers and reducing agents. **(A)** Beta-hairpin 475–486 is disordered in “5 mM Met” HSPD1 as well as in “1 mM Asc” HSPD1 **(B)**. **(C)** Additional density for Cys914 in “1 mM TCEP” β-gal and His653 rotamer is the same for “blotted” β-gal. **(D)** Cys914 remains unmodified when prepared on a continuous support film and His653 adopts the same rotamer as seen for the “fast” prepared grid and DTT added (for clarity His653 is modeled in the same rotamer position in both **(C)** and **(D)**.

The results for HSPD1 and β-gal suggested that some of the effects of prolonged exposure of proteins to the AWI or grid can be alleviated by the addition of DTT to the buffer. To better understand this observation, we tested other reducing agents which differ chemically from DTT. We prepared grids of HSPD1 by conventional blotting in the presence of 5 mM methionine or 1 mM ascorbic acid. These additives were chosen because both are known scavengers of hydroxyl radicals in aqueous solutions (Niu et al., 2015; Crosas et al., 2017). In addition, we prepared grids by conventional blotting of β-gal in the presence of 1 mM TCEP, a commonly used reducing agent which does not contain a thiol group such as DTT. We note that one of the published β-gal structures (Figure 2B) was also obtained from a sample containing TCEP (5 mM). Methionine and ascorbic acid at 5 and 1 mM, respectively, failed to protect the beta hairpin 475–486 in HSPD1 from unfolding (Figures 5A, B). The addition of 1 mM TCEP did not prevent additional density on Cys914 of β-gal (Figure 5C), and His653 adopted the same rotamer as in the modified “blotted” β-gal (Figure 5D). However, the additional density for Cys914 in 1 mM TCEP β-gal was less continuous and particles were visibly less aggregated in the raw micrographs, allowing data collection at 1 mg/ml concentration. This suggested that while relatively weak hydroxyl radical scavengers such as methionine or ascorbic acid do not protect from cysteine modification, alternative reducing agents such as TCEP might offer some protection against modification, although not as effectively as DTT did in the case of β-gal. The reasons for the difference seen between DTT and TCEP are not understood but we hope further investigations will shed further light on this observation.

To further investigate the role of the AWI we next prepared conventionally blotted grids of β-gal on grids with continuous carbon support, without reducing agent present. This produced grids with well-distributed particles showing little signs of aggregation (consistent with the rapid freezing and DTT grids). The data set provided a high-resolution structure at 2.4 Å. We saw no evidence for Cys modification for the continuous carbon grid suggesting that sequestering away from the AWI protects the cysteine residue, despite the seconds-long timescales involved in preparing the blotted grid. This suggests modification occurs at or in very close proximity to the AWI. All experimental parameters tested are shown in Table 1.

**TABLE 1:** Summary of the modifications seen in HSPD1 and β-gal and the different parameters tested. NT = not tested.

	Blotted	Fast (<50 ms)	DTT	TCEP	Carbon backed
HSPD1	Modified	No modification	No modification	Some modification	NT
β-gal	Modified	No modification	No modification	Some modification	No modification

## Discussion

While single-particle cryo-EM analysis relies on vitrifying proteins in a thin film, the full extent to which this environment impacts protein folding is yet to be fully understood. Here, we have observed spontaneous modification of cysteines during cryo-EM grid preparation, which can be prevented either by the addition of DTT, fast grid preparation, or sequestering away from the AWI. It has been proposed that in the harsh environment of the AWI, electric fields of sufficient strength are present to cause the formation of hydroxyl radicals. We speculate that hydroxyl radicals form at the AWI and react with surface-exposed cysteine residues on proteins which are located at, or close to the AWI. During the long time that the protein is exposed to the AWI in conventional blotting, this will result in the transformation of the cysteine thiol group to a sulfenic or sulfinic acid and potentially followed by additional reactions. While we think that hydroxyl radicals from the AWI are the most plausible explanation, more work is required to confirm this hypothesis. Moreover, by making a grid of continuous carbon that was also blotted we also show processes relating to the blotting paper itself are likely not responsible for the resulting cysteine modifications. It is also important to note that we observe modifications on specific cysteine residues, with others showing no modification in both β-gal and HSPD1. One explanation is that the local chemical environment of cysteine will influence its p*K*a and its sensitivity to modification. Features such as an electronegative local environment, hydrogen bond donors, and/or charged residues can lower the p*K*a significantly (Poole, 2014).

In both proteins, HSPD1 and β-gal, cysteine modification occurs together with wider structural changes. In the case of HSPD1, most of the density for beta-hairpin 475–486 is lost suggesting that this region becomes locally unfolded. In the case of β-gal, we see more subtle structural changes such as changes in rotamers, as seen for His653. We cannot unambiguously say if these structural changes are caused by the cysteine modifications observed, or whether the structural change is caused by binding to the AWI and is separate from cysteine modification. However, the fact that neither structural change is observed in the presence of DTT nor when the protein is sequestered away from the AWI suggests that cysteine modification is the cause of the structural perturbation.

This work highlights that care should be taken when interpreting the oxidation state of cysteine residues from cryo-EM structures. In addition to beam-induced effects as previously shown (Kato et al., 2021), cysteines also appear to be affected by the harsh environment of the AWI with a potential impact on the surrounding protein structure. As demonstrated for β-gal and HSPD1, cysteine modification can be prevented by the addition of a reducing agent such as DTT. In cases where reducing agents cannot be used, for example for proteins with structurally relevant disulfide bonds, fast grid preparation can be used to avoid cysteine modification, along with continuous support films. It is also interesting to note that reducing agents such as DTT has also been shown to influence the surface tension and result in increased protein aggregation and degradation (Tihonov et al., 2016). The reduction in surface tension may also mirror the effect seen with detergents which can also form a protective layer at the AWI influencing factors such as preferred orientation (Chen et al., 2022). These studies illustrate the challenges of unpicking the varying physical and chemical processes that occur at the AWI.

The above results raise the question of how commonly cysteine modification occurs during cryo-EM grid preparation. We expect that in most cases, such modifications will remain unnoticed because of limited resolution, especially in the surface-exposed regions of proteins. However, in some cases, cysteine modifications could impact protein structure. If the observed cysteine modifications are indeed caused by hydroxyl radicals from the AWI, there may also be other reactions occurring during cryo-EM grid preparation and affecting protein structure.

More work is required to understand the precise chemical processes occurring in the thin film environment. While outside the scope of this work, we have noted that there is likely a role for grid support material, as a preliminary assessment of β-gal on an UltrAuFoil^®^ grid show no modification of cysteine residues (data not shown). A future series of experiments are needed to untangle these relationships. We suggest that by understanding more precisely the impacts of the AWI on macromolecular complexes and more widely the chemistry, which is possible in the thin film environment, we will be better able to design universally successful strategies for cryo-EM sample preparation.

## Data Availability

The datasets presented in this study can be found in online repositories. All maps have been deposited within the EMDB with accession numbers; β-gal EMDB-14981 (blotted), EMDB-14982 (DTT), EMDB-14983 (TCEP), EMDB-14984 (Carbon support). HSPD1 EMDB-15178 (blotted), EMDB-15162 (fast), EMDB-11950 (blotted and DTT), EMDB-15167 (blotted with Methionine), EMDB-15176 (blotted with Ascorbate).
