# Supplementary material for: It started with a Cys: Spontaneous cysteine modification during cryo-EM grid preparation
Source: Front Mol Biosci. 2022 Aug 5;9:945772. doi: 10.3389/fmolb.2022.945772 (PMC9389043; doi:10.3389/fmolb.2022.945772)
Supplement: Supplementary file 1 [file DataSheet1.docx]

Supplementary Material

# Supplementary Data

**Supplementary Information**

*Table 1: Data collection and processing parameters for HSPD1.*

| **Data collection and processing** | **‘blotted’ HSPD1** | **‘fast’ HSPD1** | **‘5 mM Met’ HSPD1** | **‘1mM Asc’ HSPD1** |
| --- | --- | --- | --- | --- |
| Magnification | 96,000x | 96,000x | 75,000x | 75,000x |
| Detector (mode)(energy filter, if used) | Falcon IV (counting)(Selectris, 10eV) | Falcon IV (counting) Selectris, 10eV) | Falcon III (linear) | Falcon III (linear) |
| Voltage (kV) | 300 | 300 | 300 | 300 |
| Nominal defocus range (µm) | -1.5 to -3.0 | -1.8 to -3.3 | -1.5 to -3.0 | -1.5 to -3.0 |
| Pixel size (Å) | 0.82 | 0.82 | 1.07 | 1.07 |
| Total fluence (e^-^/Å^2^) | 39.9 | 56.8 | 66.7 | 64.1 |
| Number of fractions | 33 (EER) | 43 (EER) | 59 | 50 |
| Exposure time (s) | 6 | 6 | 1.5 | 1.5 |
| Number of micrographs | 2461 | 2345 | 1694 | 1254 |
| Initial number of particles | 281383 | 166797 | 178367 | 311725 |
| Final number of particles | 45334 | 5218 | 21229 | 55082 |
| Resolution (FSC = 0.143) | 3.4 | 6.8 | 4.2 | 4.8 |
| **Model Fitting** |  |  |  |  |
| Fitted model | pdb 7azp | pdb 7azp | pdb 7azp | pdb 7azp |
| Map-sharpening B-factor (Å^2^) | 0 or -100 | -100 | 0 or -100 | -100 |

*Table 2: Data collection and processing parameters for β-gal.*

| **Data collection and processing** | **Fast'** | **Blotted'** | **DTT'** | **TCEP'** | **Continuous support'** |
| --- | --- | --- | --- | --- | --- |
| Magnification | 165,000 x | 96,000 x | 96,000 x | 96,000 x | 96,000 x |
| Detector (mode)  (energy filter, if used) |  | Falcon IV (counting)  No energy filter | Falcon IV (counting)  No energy filter | Falcon IV (counting)  No energy filter | Falcon IV (counting)  No energy filter |
| Voltage (kV) | 300 | 300 | 300 | 300 | 300 |
| Nominal defocus range (µm) | -1.0 to -2.5 | -0.8 to -2.0 | -0.8 to -2.0 | -0.8 to -2.0 | -0.6 to -2.4 |
| Pixel size (Å) | 0.73 | 0.86 | 0.86 | 0.86 | 0.86 |
| Total fluence (e^-^/Å^2^) | 38.1 | 37.9 | 37.9 | 37.9 | 34.86 |
| Number of fractions | 38 (EER) | 38 (EER) | 38 (EER) | 38 (EER) | 44 (EER) |
| Exposure time (s) | 3 | 3.98 | 3.98 | 3.98 | 5.82 |
| Number of micrographs | 2556 | 2887 | 1084 | 1121 | 5941 |
| Initial number of particles | 110515 | 113073 | 224882 | 160168 | 100000 |
| Final number of particles | 30363 | 77517 | 129845 | 82807 | 51462 |
| Resolution (FSC = 0.143) | 2.4 | 2.2 | 2.1 | 2.4 | 2.4 |
| **Model Fitting** |  |  |  |  |  |
| Fitted model | Pdb 6tte | pdb 6CVM | pdb 6CVM | pdb 6CVM | pdb 6CVM |
| Map-sharpening B-factor (Å^2^) | 0 or -38 | 0 or -39 | 0 or -37 | 0 or -59 | 0 or -42 |


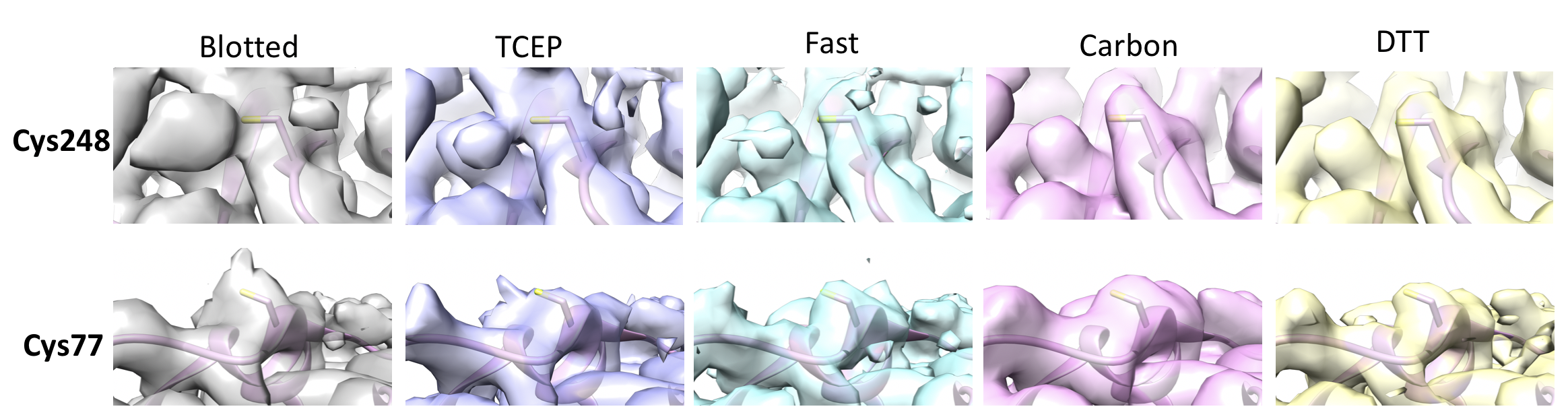
Supplementary Figure 1. Cys modification also present on residues 248 and 77.


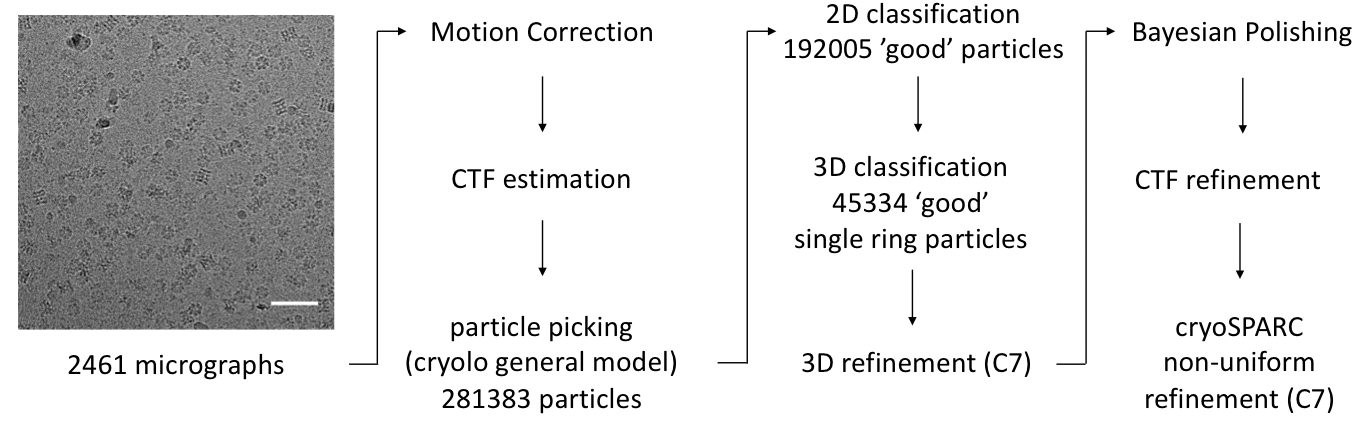


*Supplementary Figure 2: Data processing pipeline for ‘blotted’ HSPD1 data processing.*

*Supplementary Figure 3: Data processing pipeline for ‘fast’ HSPD1 data processing.*

**

*Supplementary Figure 4: Data processing pipeline for ‘5 mM Met’ HSPD1 data processing.*

**

*Supplementary Figure 5: Data processing pipeline for ‘1 mM Asc’ HSPD1 data processing.*

2887

m

i

c

r

o

g

r

a

p

h

s

M

o

t

i

o

n

C

o

r

r

e

c

t

i

o

n

C

T

F

e

s

t

i

m

a

t

i

o

n

p

a

r

t

i

c

l

e

p

i

c

k

i

n

g

(

c

r

y

o

l

o

g

e

n

e

r

a

l

m

o

d

e

l

)

113073

p

a

r

t

i

c

l

e

s

2

D

c

l

a

s

s

i

f

i

c

a

t

i

o

n

84623

’

g

o

o

d

’

p

a

r

t

i

c

l

e

s

3

D

c

l

a

s

s

i

f

i

c

a

t

i

o

n

77517

‘

g

o

o

d

’

s

i

n

g

l

e

r

i

n

g

p

a

r

t

i

c

l

e

s

3

D

r

e

f

i

n

e

m

e

n

t

(

D2

)

CTF refinement

Bayesian polishing


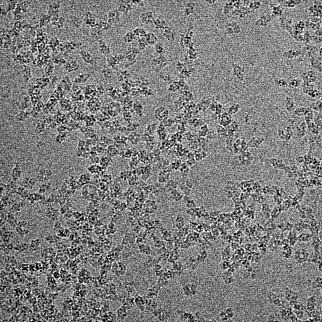


*Supplementary Figure 6: Data processing pipeline for ‘blotted’ β-gal*

1084

m

i

c

r

o

g

r

a

p

h

s

M

o

t

i

o

n

C

o

r

r

e

c

t

i

o

n

C

T

F

e

s

t

i

m

a

t

i

o

n

p

a

r

t

i

c

l

e

p

i

c

k

i

n

g

(

c

r

y

o

l

o

g

e

n

e

r

a

l

m

o

d

e

l

)

224882

p

a

r

t

i

c

l

e

s

2

D

c

l

a

s

s

i

f

i

c

a

t

i

o

n

163056

’

g

o

o

d

’

p

a

r

t

i

c

l

e

s

3

D

c

l

a

s

s

i

f

i

c

a

t

i

o

n

129845

‘

g

o

o

d

’

s

i

n

g

l

e

r

i

n

g

p

a

r

t

i

c

l

e

s

3

D

r

e

f

i

n

e

m

e

n

t

(

D2

)

CTF refinement

Bayesian polishing


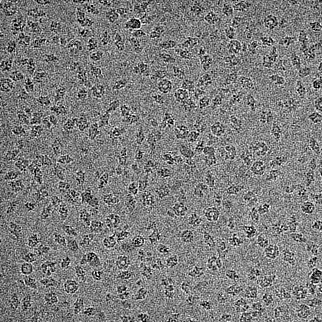


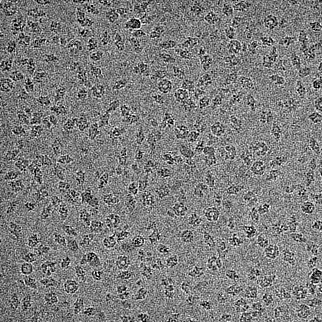
*Supplementary Figure7: Data processing pipeline for ‘1 mM DTT’ β-gal*


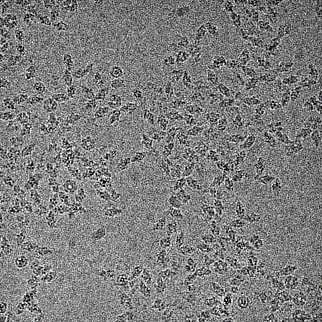
*
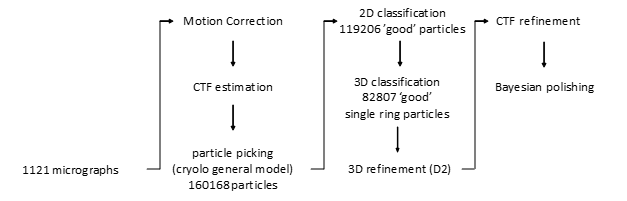
*

*Supplementary Figure 8: Data processing pipeline for ‘1 mM TCEP’ β-gal*

5941

m

i

c

r

o

g

r

a

p

h

s

M

o

t

i

o

n

C

o

r

r

e

c

t

i

o

n

C

T

F

e

s

t

i

m

a

t

i

o

n

p

a

r

t

i

c

l

e

p

i

c

k

i

n

g

(

c

r

y

o

l

o

g

e

n

e

r

a

l

m

o

d

e

l

)

100000

p

a

r

t

i

c

l

e

s

2

D

c

l

a

s

s

i

f

i

c

a

t

i

o

n

56377

’

g

o

o

d

’

p

a

r

t

i

c

l

e

s

3

D

c

l

a

s

s

i

f

i

c

a

t

i

o

n

51462

‘

g

o

o

d

’

s

i

n

g

l

e

r

i

n

g

p

a

r

t

i

c

l

e

s

3

D

r

e

f

i

n

e

m

e

n

t

(

D2

)

CTF refinement

Bayesian polishing


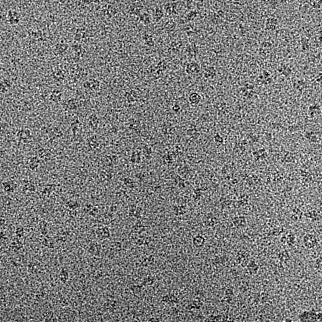


*Supplementary Figure 9: Data processing pipeline for ‘continuous support’ blotted β-gal*
